# Experiences of Female Informal Traders With Access to Health Care Services in Africa: Protocol for a Scoping Review

**DOI:** 10.2196/89997

**Published:** 2026-09-09

**Authors:** Gugulethu Moonlight Zondi, Senzo Hopewell Mpangase, Esther Lydie Mbobnda Kapche, Firoza Haffejee

**Affiliations:** 1 Basic Medical Sciences Durban University of Technology Durban, KwaZulu-Natal South Africa

**Keywords:** informal traders, women, female, access to health care services, Africa, women’s health

## Abstract

**Background:**

The African continent has witnessed a notable shift in its economic landscape, marked by a significant rise in unemployment and a parallel surge in informal trading activities. In response to economic challenges, a substantial number of women within the informal trading sector in African countries have ventured beyond their home settings to engage in trading, aiming to alleviate poverty within their households. However, this pursuit is often characterized by conditions of poverty and socioeconomic difficulties. These women frequently lack access to fundamental health care and social protection services, and they operate within environments that are suboptimal for health and safety. This highlights a gap in knowledge regarding their challenges and barriers in accessing health care and an opportunity for better understanding.

**Objective:**

This scoping review aims to synthesize the existing evidence related to the accessibility of health care services for women who engage in informal trade.

**Methods:**

This scoping review will adhere to the methodological guidelines outlined in the JBI framework, and the results will be presented in accordance with the PRISMA-ScR (Preferred Reporting Items for Systematic Reviews and Meta-Analyses Extension for Scoping Reviews) guidelines. Eligibility criteria will include studies focusing on women engaged in informal trading as the primary population of interest, articles published in English or French, and studies conducted in African countries. To achieve this, relevant scholarly literature will be identified through comprehensive keyword-based searches across several databases, including PubMed, Scopus, CINAHL, Web of Science, EBSCOhost, and SA ePublications, spanning from January 1995 to June 2026. The review’s focus will be constrained to research evidence originating in Africa. All identified records will be collated and uploaded into EndNote X25 for management. Eligible studies will be screened by 2 independent reviewers, and disagreements will be resolved through discussion and the intervention of a third reviewer. Data will be independently extracted by 2 reviewers using a validated data extraction form developed for this review and will be analyzed using content analysis. The findings will be presented in a narrative format.

**Results:**

The initial database search was completed in October 2025 and updated in June 2026. The review is currently in the screening stage. The review is expected to be completed by October 2026.

**Conclusions:**

The scoping review aims to identify and map the evidence regarding the physical, financial, cultural, and social barriers that female informal traders in Africa face in accessing health care services, particularly financial constraints that may limit their enrollment in national health insurance. The findings will offer insights into these challenges, helping to inform future strategies to improve health care access and enhance the well-being of female informal traders.

**Trial Registration:**

OSF Registries 10.17605/OSF.IO/V9KJX; https://osf.io/v9kjx/overview

**International Registered Report Identifier (IRRID):**

PRR1-10.2196/89997

## Introduction

### Background

Access to quality, affordable health care is central to the achievement of health equity and is a core objective of the global universal health coverage (UHC) agenda. Despite progress in some settings, many health systems do not meet UHC targets, and informal workers, in particular, are frequently excluded from health protection mechanisms and primary care services [1]. The concept of the “informal sector,” first articulated by Hart [2] in the early 1970s, has evolved to encompass a wide range of economic activities not captured by formal regulatory frameworks. Informal trading typically refers to various income-generating activities conducted outside formal labor markets. These include street vending, market stalls, and home-based businesses [3]. Moreover, the informal sector is characterized by unskilled labor, little job security, and no fringe benefits [4]. Research has shown that people turn to informal trading because of poverty, low education, large family sizes, emigration from rural areas, and the need to provide for the household [5,6].

Globally, an estimated 60% of women participate in the informal economy, with the proportion rising to more than 90% in low-income regions such as Southern Asia and sub-Saharan Africa [7,8]. Furthermore, the International Labour Organization indicates that approximately 20% of the global workforce makes substantial contributions to the global gross domestic product through their participation in informal sector activities [9]. Workers in the informal sector are either self-employed or employed by other self-employed people. Self-employed people are typically not on a payroll and are therefore not subject to taxes; hence, they may operate in unsafe and insecure locations [10]. Informal workers typically lack access to essential benefits such as paid leave and health care services [7].

Despite the substantial representation of women in this sector, informal trading is frequently associated with poverty and heightened vulnerability [11,12]. Female workers often contend with precarious employment conditions, inadequate wages, and substandard working environments, while simultaneously managing domestic responsibilities that further restrict their opportunities [13]. Female informal traders face deeply interconnected social and health challenges shaped by systemic exclusion and the unregulated nature of the informal economy. Socially, these women contend with limited access to finance, secure trading spaces, and legal recognition, which restrict their ability to grow sustainable businesses [14]. Gender-specific vulnerabilities, including harassment, violence, unsafe working conditions, and regulatory neglect, further marginalize them [12,15,16]. Furthermore, structural barriers such as restrictive municipal bylaws and inadequate infrastructure disproportionately affect women. The municipal bylaws, such as permit requirements and location limitations, further limit their activities [17,18].

Female informal traders are also exposed to hazardous, unhygienic, and poorly regulated working environments, combined with a lack of access to clean water, sanitation, and safety infrastructure, which contribute to a high incidence of illness and injury [16,19,20]. Reports of unsafe workspaces, prolonged working hours, and exposure to physical and chemical hazards are common, with studies linking these conditions to serious health problems such as dizziness, skin burns, and chronic fatigue [21-23]. In Ghana, research findings highlighted a substantial lack of national health insurance among women informal traders, further intensifying their challenges in accessing health care services [24].

While the literature provides a descriptive account of informal trading, particularly street vending [25,26], it tends to focus more on the economic dimensions and less on social and health analyses. However, a substantial proportion of female informal traders are young women of reproductive age and older women living with underlying chronic conditions. Consequently, the urgency of health care access among this population becomes evident. Good health is closely linked to their livelihoods, and any disruption can have far-reaching economic consequences. In the context of these economic realities, female informal traders are particularly vulnerable due to the absence of national health insurance and their reliance on public health care services.

Despite studies conducted on informal trading, as of November 2025, a preliminary search of the PROSPERO database, the Cochrane Database of Systematic Reviews, JBI Evidence Synthesis, and the Open Science Framework indicates that there are no previous or ongoing scoping reviews that summarize the experiences and challenges of female informal traders with regard to access to health care in African countries. A recent review of health and informal trading focused on health outcomes among mothers and children in both high-income countries and low- and middle-income countries [27]. Another review of informal employment and health equity globally did not have an explicit gender perspective specific to African countries, where household and reproductive responsibilities, combined with poverty, mostly drive women into flexible, low-risk economic activities [28]. However, informality is of particular concern for women [29]. Therefore, the proposed scoping review will explore the experiences and challenges of female informal traders in Africa, with a focus on their access to health care.

### Review Questions

The primary research question of this scoping review is as follows: “What is the extent and nature of existing research on the access to health care services for women engaged in informal trading?” Subsidiary questions include the following:

- Do female informal traders experience unhindered access to health care services?
- What are the key challenges and contributing factors blocking female informal traders’ access to health care services?
- What research studies have been conducted within the African context?

## Methods

The proposed scoping review will be conducted according to the JBI methodology for scoping reviews and adhere to the PRISMA-ScR (Preferred Reporting Items for Systematic Reviews and Meta-Analyses Extension for Scoping Reviews) guidelines [30].

### Registration

This review is registered with the Open Science Framework repository [31].

### Eligibility Criteria

#### Overview

Eligibility criteria for this scoping review will draw from the JBI mnemonic for the formulation of scoping review questions, which describes the population, concept, and context of the study [32]. For studies to be included, they should meet the following criteria: be available in full text in English or French, be related to female informal traders, focus on access to health care, and be published between January 1995 and June 2026. The time frame beginning January 1995 was selected to represent the period following major socioeconomic and policy transitions that influenced the development and regulation of the informal economy in many African contexts, including urban informal trading structures. This time frame allows the inclusion of contemporary and policy-relevant literature while maintaining a manageable scope for the synthesis. Studies will be excluded if the full text is not available, they focus on male informal traders or women engaged in formal employment, they do not address access to health care, they fall outside the predefined search period, or they are not available in English or French. Textbox 1 summarizes the inclusion and exclusion criteria.

**Inclusion and exclusion criteria.**

**Inclusion criteria**

- Population: studies conducted on women who are involved in informal trading activities, such as street vending, market stalls, home-based businesses, or any other form of informal economic activity
- Concept: studies on access to health care
- Context: studies conducted in Africa
- Time frame: studies conducted from January 1995 to June 2026
- Language: studies published in English or French

**Exclusion criteria**

- Population: studies conducted on women involved in any formal trading activities or any informal nontrading activities, and studies conducted on male informal traders
- Concept: studies conducted on any other aspect of health care
- Context: studies conducted outside of Africa
- Time frame: studies conducted before 1995
- Language: studies published in any other language apart from English or French

#### Population

For this scoping review, we will source all relevant peer-reviewed articles regarding women involved in any informal trading activity. We will exclude studies on women involved in any formal trading activity or in any informal, nontrading activity. We will also exclude studies on male informal traders.

#### Concept

This review is centered on the intersection of women’s engagement in informal trading and their access to health care services. It involves examining the various aspects related to how women who are involved in informal trading interact with health care systems, including the barriers they face, the factors that enable or hinder access, and the potential solutions or interventions to improve their access to health care. The concept focuses on issues related to access to health care. Any studies or data that do not directly address or contribute to understanding the health care access challenges faced by women engaged in informal trading will be excluded.

#### Context

The context for this scoping review is Africa, which was selected because of the widespread prevalence of informal trading activities. It acknowledges that women engaged in informal trading operate within a diverse range of cultural and subcultural backgrounds, where traditional beliefs and societal norms may influence health care–seeking behaviors. The geographic locations where informal trading occurs are varied, spanning urban and rural settings and crossing national boundaries. This diversity is vital as it affects health care accessibility, service availability, and infrastructure. The specific setting details, such as street markets, home-based businesses, or informal vending sites, are integral to understanding the environmental and logistical factors that influence access to health care within the context of informal trading.

#### Time Frame

Eligible studies conducted from January 1995 to June 2026 will be included.

### Types of Sources

We will seek all peer-reviewed literature related to the study objectives. We will include gray literature consisting of government reports, book chapters, conference proceedings, policy documents, government publications, theses, and dissertations, as well as publications from recognized nongovernmental organizations and research institutions working on the social and health challenges of female informal traders. Only materials clearly identifying authors, publication dates, and institutional sources will be reviewed. Sources lacking methodological detail, unpublished opinion articles, blog posts, news items, newsletters, conference abstracts without full reports, and documents from untrustworthy sources will be excluded. We will consider experimental and quasi-experimental study designs, observational studies, and qualitative studies. We will also conduct a hand search of the reference lists of all included articles to identify any additional relevant studies.

### Search Strategy

A 3-step search strategy will be used in this review. First, an initial limited search was conducted in the PubMed database to examine the words in the title and abstract, index terms, and keywords of several papers and identify search terms (Multimedia Appendix 1). Thereafter, the list of search terms was devised and reviewed by the librarian at the Durban University of Technology to develop a full search strategy for the PubMed database. Six electronic databases (PubMed, Scopus, CINAHL, Web of Science, EBSCOhost, and SA ePublications) were systematically searched. The search strategy will be adapted for each included database or information source. Peer-reviewed journals will be reviewed for primary studies with a clear empirical base using qualitative, quantitative, and mixed methods addressing the research questions. In addition, we will search for articles through the “cited by” search. Once the article review is completed, we will search the reference lists of all the included articles to identify additional articles.

The university’s interlibrary loan system will be used to obtain the full texts of articles from journals that the university does not subscribe to. Furthermore, an email and a reminder will be sent to the corresponding authors of articles whose full texts are not available. If there is no response after a reasonable time, the articles will be excluded from the review. The first search was conducted in October 2025, and an updated search was conducted in June 2026.

The following keywords and Boolean operators will be combined in the search strategy: “Women” OR “Woman” OR “female” AND “Informal trading” OR “street trading” OR “spaza shops” OR “tuck shop” OR “street food vendors” OR “clothing vendors” OR “fruit vendors” OR “vegetable vendors” OR “shoe repairs” OR “informal economy” OR “House shop” OR huiswinkel OR “informal sector” OR “small business” OR vendor* AND “Health care” OR “access to health service*” OR “health services” OR clinics OR hospital* OR “medical centre” OR “public health care” OR “public healthcare” AND Africa OR Nigeria OR “South Africa” OR Kenya OR Sudan OR Ghana OR Morocco OR Congo OR Senegal OR Tanzania OR Uganda OR Algeria OR “Cote d’Ivoire” OR Somalia OR Madagascar OR Niger OR Angola OR Zimbabwe OR Rwanda OR Namibia OR Gabon OR Burkina Faso OR Zambia OR South Sudan OR Mozambique OR Guinea OR Chad OR Liberia OR Togo OR Libya OR “Central African Republic” OR Djibouti OR Burundi OR Malawi OR Lesotho OR Benin OR Mauritius OR Eritrea OR “Sierra Leone” OR Mauritania OR Eswatini OR Swaziland OR Seychelles OR Gambia. The search strategy was piloted in the PubMed database to check the appropriateness of selected electronic databases and keywords. An experienced information specialist ensured that a robust review search strategy was followed. Boolean terms (AND, OR, and NOT) were used to separate the keywords. Additionally, wildcards and truncations were used to accommodate variations in the different databases. All relevant evidence sources were identified, compiled in EndNote X25 reference manager (Clarivate Analytics), and exported to the Rayyan systematic review software (Qatar Computing Research Institute) for screening. Duplicates will be removed and recorded before the screening starts.

### Study and Source of Evidence Selection

**Figure 1:**
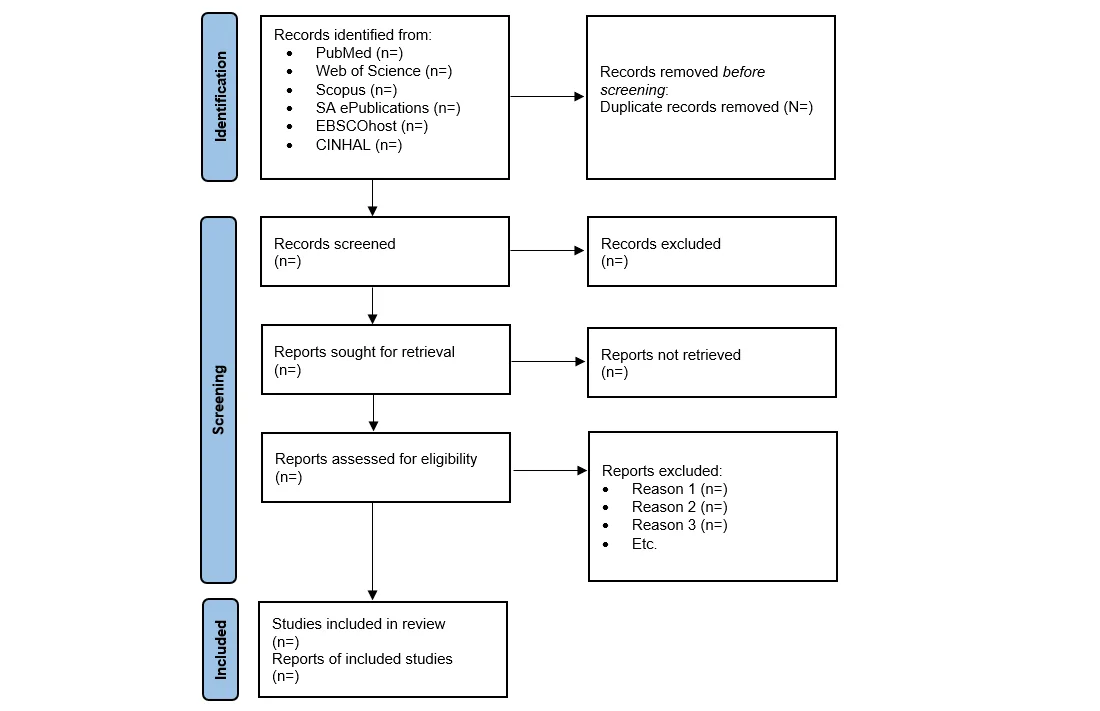
PRISMA (Preferred Reporting Item for Systematic Reviews and Meta-Analyses) flowchart.

The titles and abstracts will be independently screened using Rayyan according to the eligibility criteria. Thereafter, potentially relevant sources will be retrieved in full and will be assessed independently against the inclusion criteria. The following screening instructions were shared with the 2 screeners: “You are required to systematically screen the titles, abstracts, and full texts of eligible studies to determine their relevance based on the predefined inclusion and exclusion criteria. Please familiarise yourself with the eligibility criteria. If there is any doubt in any screening stage, please include the study for further screening. A study should be included if it meets all the inclusion criteria and none of the exclusion criteria. A study should be excluded if it fails to meet one or more of the inclusion criteria or if it meets one or more of the exclusion criteria. Document your screening decisions (include/exclude/maybe) and provide a brief reason for each decision.” The reasons for excluding a reference will be documented. During screening, journal names, authors, and publication years will not be hidden from screeners. Any disagreements that arise between the reviewers at each stage of this process will be resolved through discussion until a consensus is reached and/or with the help of a third reviewer. To minimize bias during the screening rounds, the 2 authors will independently screen all sources, and a coefficient of agreement will be calculated at each round. If the coefficient is <50%, the 2 screeners will discuss the eligibility criteria to obtain a better understanding. The results of the search and the study inclusion process will be reported in a PRISMA-ScR flow diagram (Figure 1) [33]*.*

### Quality Appraisal

The quality of all full-text peer-reviewed studies included in this review will be independently appraised by 2 reviewers using the Mixed Methods Appraisal Tool (version 2018) [34] by rating each item using “yes,” “no,” or “can’t tell.” The Mixed Methods Appraisal Tool was chosen because it allows for the appraisal of qualitative, quantitative, and mixed methods studies using the same tool [34].

### Data Extraction

Data from papers included in the scoping review will be independently extracted by 2 reviewers using a data extraction tool on Google Forms developed by the reviewers (Multimedia Appendix 2). The tool will be pilot-tested on 5 articles and will be modified if the extraction alignment is poor or if changes are needed. All modifications will be documented. Data will be synthesized using a descriptive analytical approach that adheres to the scoping review methodology. The extracted data will be charted and organized based on the review’s research questions. The findings will be organized to address (1) the types of social challenges faced by female informal traders, (2) the health challenges reported, (3) the structural and contextual factors (including policy and infrastructure) that influence these challenges, and (4) gaps in the existing literature. A narrative summary will accompany the tabulated results, describing patterns, similarities, and variations. Specific details such as (1) metadata (authors, year of publication, and country), (2) study methods and sample size, (3) challenges, (4) experiences, (5) barriers, and (6) any other significant findings will be extracted. The following instructions will be shared with the 2 reviewers before data extraction: “Carefully extract all relevant data from each included study, as outlined in the extraction form. Ensure that all required data points are extracted, even if they are not immediately obvious. If any data are missing, clearly document that in the extraction form. Always indicate the source of the data (specific paragraph, table, figure).” The Microsoft Excel spreadsheet outputs will be shared among the authors for discussion and validation before data synthesis. Finally, a Cohen κ coefficient will be calculated to measure the agreement between the 2 data extractors. A κ value of 1 will indicate perfect agreement, whereas a value of 0 will indicate an agreement no better than chance.

### Ethical Considerations

Ethics approval is not required, as the review is a synthesis of published literature.

### Data Analysis and Presentation

All the authors will be involved in the synthesis. One author is a native French speaker and will read and understand all eligible studies published in French. A descriptive approach using thematic analysis [35] for data summarization and reporting will be adopted. The findings of the included studies related to the experiences and challenges encountered by female informal traders in accessing health care services will be presented in a narrative form to provide a more holistic interpretation of the findings. It is anticipated that eligible studies will mainly be qualitative. Therefore, a meta-analysis would be unsuitable for our review. The data extraction tool will be modified as necessary during the charting process to guarantee relevance and clarity. To improve impartiality and reliability, 2 reviewers will independently extract data. Reviewers will not be blinded to study authors, journal titles, publication years, or other identifying information, as this information is necessary for citation management and data extraction. Any differences will be handled by discussion or consultation with a third reviewer.

## Results

The scoping review is progressing according to the following timeline. The development and registration of the review protocol were completed in December 2025. Initial database searches commenced in October 2025 and were completed in June 2026. Screening of titles and abstracts was performed in January 2026, with full-text review and data extraction expected to be completed in July 2026. Data synthesis and analysis will be conducted in August 2026, and final manuscript preparation is anticipated by October 2026. Preliminary findings indicate a broad and varied body of literature on women in informal trading, particularly in relation to barriers and facilitators affecting their access to health care services. The next steps will involve identifying relevant studies through systematic screening of titles and abstracts.

## Discussion

### Anticipated Contribution of the Review

In June 2026, the updated database search identified 485 articles from the PubMed, Scopus, CINAHL, Web of Science, EBSCOhost, and SA ePublications databases. The subsequent phase will be the retrieval of eligible gray literature. This scoping review aims to provide a comprehensive overview of the current evidence on access to health care services among women engaged in informal trading. By mapping existing literature, the review seeks to (1) inform policymakers, health care providers, and researchers about the specific health care access challenges faced by this population; (2) identify innovative and community-based strategies that have been used to improve health care access; (3) explore the barriers and facilitators influencing health care use among female informal traders; and (4) highlight gaps in the literature to guide future research and targeted interventions. Ultimately, the findings will support the development of more inclusive and responsive health care services tailored to the needs of women engaged in the informal economy.

### Potential Implications

The outcomes of this scoping review are expected to offer valuable insights that can inform health care practice, policy, and education, particularly in the context of underserved populations such as women engaged in informal trading. By identifying existing barriers and facilitators to health care access, the review may guide the development of context-sensitive interventions and policies that are more responsive to the lived experiences of female informal traders. Additionally, the findings may contribute to the enhancement of community health education and training programs by promoting inclusive, grassroots-informed strategies. This could ultimately influence curriculum development, shape public health outreach, and inform decision-making at both institutional and policy levels, ensuring that the specific needs of this vulnerable group are more effectively addressed within health care systems.

### Strengths and Limitations

This scoping review is strengthened by its comprehensive and systematic search strategy, which aims to capture a broad range of relevant literature on access to health care services among women engaged in informal trading. By considering studies published in English and French, the review also enhances inclusivity and reduces language bias. A key strength of this review lies in its focus on a relatively underresearched area, shedding light on the health care access challenges faced by a marginalized and often overlooked population group. However, certain limitations may arise, including the risk of missing relevant studies not available in the selected languages or those published in gray literature sources. Additionally, synthesizing diverse forms of evidence across varied geographical and socioeconomic contexts may present analytical challenges. Despite these limitations, the review is positioned to provide valuable insights that can inform policy, health system responsiveness, and future research priorities related to health care access among women engaged in informal trading.

### Conclusions

This scoping review aims to offer an explicitly gendered perspective on informal trading and access to health care services. By systematically mapping the current body of literature, the review will highlight existing strategies, identify barriers and facilitators, and expose gaps in knowledge related to health care access among this population, particularly in relation to experiences of discriminatory practices. The findings are expected to inform future research, guide health policy, and support the development of targeted interventions to improve health care access for women working in the informal economy.

## Appendix

Multimedia Appendix 1 Pilot search in the PubMed database.

Multimedia Appendix 2 Data extraction form.

Multimedia Appendix 3 PRISMA-Scr checklist.

## Abbreviations

PRISMA-ScR: Preferred Reporting Items for Systematic Reviews and Meta-Analyses Extension for Scoping Reviews
UHC: universal health coverage

## Data Availability

All data generated and analyzed during this study will be included in the published scoping review article and will be available from the corresponding author on reasonable request.
