# Supplementary material for: Experiences of Female Informal Traders With Access to Health Care Services in Africa: Protocol for a Scoping Review
Source: JMIR Res Protoc. 2026 Sep 9;15:e89997. doi: 10.2196/89997 (PMC13601873; doi:10.2196/89997)
Supplement: Supplementary file 2 [file resprot_v15i1e89997_app1.docx]

**Multimedia Appendix 1: Pilot search in PubMed**

(((Women[Text Word] OR Woman[Text Word] OR female[Text Word]) AND ("Informal trading"[Text Word] OR "street trading"[Text Word] OR "spaza shops"[Text Word] OR "tuck shop"[Text Word] OR "street food vendors"[Text Word] OR "clothing vendors"[Text Word] OR "fruit vendors"[Text Word] OR "vegetable vendors"[Text Word] OR "shoe repairs"[Text Word] OR "informal economy"[Text Word] OR "House shop"[Text Word] OR huiswinkel[Text Word] OR "informal sector"[Text Word] OR "small business"[Text Word] OR vendor*[Text Word])) AND ("Health care"[Text Word] OR "access to health service*"[Text Word] OR "health services"[Text Word] OR clinics[Text Word] OR hospital*[Text Word] OR "medical centre"[Text Word] OR "public health care"[Text Word] OR "public healthcare"[Text Word])) AND (Africa[Text Word] OR Nigeria[Text Word] OR "South Africa"[Text Word] OR Kenya[Text Word] OR Sudan[Text Word] OR Ghana[Text Word] OR Morocco[Text Word] OR Congo[Text Word] OR Senegal[Text Word] OR Tanzania[Text Word] OR Uganda[Text Word] OR Algeria[Text Word] OR "Cote d’Ivoire"[Text Word] OR Somalia[Text Word] OR Madagascar[Text Word] OR Niger[Text Word] OR Angola[Text Word] OR Zimbabwe[Text Word] OR Rwanda[Text Word] OR Namibia[Text Word] OR Gabon[Text Word] OR Burkina Faso[Text Word] OR Zambia[Text Word] OR South Sudan[Text Word] OR Mozambique[Text Word] OR Guinea[Text Word] OR Chad[Text Word] OR Liberia[Text Word] OR Togo[Text Word] OR Libya[Text Word] OR "Central African Republic"[Text Word] OR Djibouti[Text Word] OR Burundi[Text Word] OR Malawi[Text Word] OR Lesotho[Text Word] OR Benin[Text Word] OR Mauritius[Text Word] OR Eritrea[Text Word] OR "Sierra Leone"[Text Word] OR Mauritania[Text Word] OR Eswatini[Text Word] OR Swaziland[Text Word] OR Seychelles[Text Word] OR Gambia[Text Word]) Filters: English, French, Female, from 1995/1/1 - 2025/9/30
