# Supplementary material for: Experiences of Female Informal Traders With Access to Health Care Services in Africa: Protocol for a Scoping Review
Source: JMIR Res Protoc. 2026 Sep 9;15:e89997. doi: 10.2196/89997 (PMC13601873; doi:10.2196/89997)
Supplement: Supplementary file 3 [file resprot_v15i1e89997_app2.docx]

**Multimedia Appendix 2: Data extraction form**

Article authors and date

Country of origin

Study aim

Study design (quantitative, qualitative, mixed)

Population

Sample size

Study setting

Types of social challenges faced by female informal traders

Health challenges reported

Structural and contextual influencing factors

Gaps in the existing literature

Unintended outcomes (something they were not looking for)

Conclusion
